# Identification and characteristics of extracellular vesicles from bovine blastocysts produced *in vitro*

**DOI:** 10.1371/journal.pone.0178306

**Published:** 2017-05-25

**Authors:** Edwin A. Mellisho, Alejandra E. Velásquez, María J. Nuñez, Joel G. Cabezas, Juan A. Cueto, Claudio Fader, Fidel O. Castro, Lleretny Rodríguez-Álvarez

**Affiliations:** 1 Laboratory of Animal Biotechnology, Department of Animal Science, Faculty of Veterinary Science, University of Concepción, Chillán, Concepción, Chile; 2 Laboratory of Cell and Molecular Biology, IHEM-CONICET, Faculty of Medical Sciences, National University of Cuyo, Mendoza, Argentina; Justus Liebig Universitat Giessen, GERMANY

## Abstract

Extracellular vesicles (EVs) have been identified within different body fluids and cell culture media. However, there is very little information on the secretion of these vesicles during early embryonic development. The aims of this work were first to demonstrate the secretion of extracellular vesicles by pre-implantation bovine embryos and second to identify and characterize the population of EVs secreted by bovine blastocysts during the period from day seven to nine of embryo culture and its correlation with further embryo development up to day 11. Bovine embryos were produced by in vitro fertilization (IVF) or parthenogenetic activation (PA) and cultured until blastocyst stage. Blastocyst selection was performed at day 7 post IVF/PA considering two variables: stage of development and quality of embryos. Selected blastocysts were cultured in vitro for 48 hours in groups (exp. 1) or individually (exp. 2) in SOF media depleted of exosomes. At day 9 post IVF/PA the media was collected and EVs isolated by ultracentrifugation. Transmission electron microscopy revealed the presence of heterogeneous vesicles of different sizes and population: microvesicles (MVs) and exosomes (EXs) of rounded shape, enclosed by a lipid bi-layer and ranging from 30 to 385 nm of diameter. Flow cytometry analysis allowed identifying CD63 and CD9 proteins as exosome markers. Nanoparticle tracking analysis generated a large number of variables, which required the use of multivariate statistics. The results indicated that the concentration of vesicles is higher in those blastocysts with arrested development from day 9 up to day 11 of in vitro development (6.7 x 10^8^ particles/ml) derived from IVF (p <0.05), compared to PA blastocysts (4.7 x 10^8^ particles/ml). Likewise, the profile (concentration and diameter) of particles secreted by embryos derived from IVF were different from those secreted by PA embryos. In conclusion, we demonstrated that bovine blastocysts secrete MVs/EXs to the culture media. Data suggest that characteristics of the population of EVs vary depending on embryo competence.

## Introduction

There is increasing evidence that EVs secreted by cells play important roles in cell-cell communication and they can be identified in vivo in different biological fluids, including blood, urine, as well as in vitro in cell culture media [1,2]. EVs are related to biological events including tumorigenesis, metabolism, coagulation, intercellular communication and the immune system [3] and they also have multiple applications in diagnosis and therapies of different pathologies [4].

There are three groups of EVs that can be identified by their size and shape: microvesicles (MV) are the biggest vesicles (diameter: 100–1000 nm) followed by apoptotic bodies (AB: 50–500 nm) and, exosomes (EXs), the smallest vesicles with a range size of 30–120 nm [5]. The populations of EVs secreted by cells vary in size and concentration and are heterogeneous in their cellular origin [6,7]. For that reason, the International Society for Extracellular Vesicles (ISEV) suggested to include in publications an overview of the protein composition of purified vesicles, especially transmembrane proteins (tetraspanins CD9, CD63, CD81) and cytosolic proteins with membrane-binding capacity (TSG101, annexins = ANXA, Rabs = RAB) [8]. However, morphological identification and characterization requires several technologies such as transmission electronic microcopy (TEM), flow cytometry (FACS) and nanoparticle tracking analysis (NTA) [1,9].

Both MVs and EXs work as carriers of different types of molecules such as proteins, lipids, messenger RNAs (mRNA) and non-coding RNAs (miRNAs) [10]. Because of this functionality, it seems that MVs/Exo secretion is a mechanism to assure cellular communication. Within the MVs/EXs, the signaling molecules are protected against degradation or inactivation in the extracellular environment, conserving their three dimensional structure and, therefore, their biological activity [11].

Embryo development is a complex process that depends on the bidirectional interaction of the embryo with the maternal environment. As well as in other biological process, MVs/EXs might mediate embryo-maternal cross-talk [12]. In 2014, Saadeldin et al. [13] identified MVs/EXs secreted from porcine parthenogenetic embryos through TEM and revealed a possible mechanism of paracrine communication between cells during early embryonic development [13]. However, there is very little information of the secretion of these vesicles during early embryonic development and whether secretion may be affected by embryo quality [14] and state of its development [13]. The presence of the zona pellucida (ZP) may modulate the secretion of EVs of different sizes.

The current consensus is that autophagy is the primary protector of cell death, that contributes to increase cell survival, presumably through the selective removal of late endosomes by autophagy machinery [15,16] and re-routing the multivesicular bodies (MVB) towards the autophagy pathway, although these mechanisms obstruct exosome release [17]. Thus, we hypothesized that the secretion of extracellular vesicles could indicate cell status and be useful for selecting competent blastocyst.

The goals of this study were: 1) to perform morphological identification of EVs secreted by bovine blastocysts and 2) to determine a possible correlation between population and characteristics of EVs and embryo competence. Based on that, we report for the first time, the morphological identification of EVs secreted by bovine blastocysts. We also identified MVB that were originated in the intracellular compartments of the blastomeres. Using nanoparticle tracking analysis and flow cytometry, we identified and characterized the population of EVs secreted to the culture media from day 7 to 9 of embryo development and correlated it with blastocysts developmental competence in vitro.

## Materials and methods

All experiments were approved by the Ethics Committee of the Faculty of Veterinary Sciences, University of Concepcion under the granting number CBE-28-15.

### Experimental design

To accomplish the proposed goals, two experiments were conducted. The first experiment was aimed to identify the presence of EVs in the culture media from pre-implantation bovine embryos produced by IVF and PA and also to identify structures associated with EVs biogenesis in the intracellular compartments of the blastomeres. It was presumed that due to the small number of cells in a single blastocysts, there will be as well little quantity of secreted EVs, and thus TEM determination would be hampered. To avoid this, we conducted this experiment using pools of 10 blastocysts. For this, Day-7 blastocysts were selected and cultured for two more days, up to day 9, in groups of ten in SOF media depleted of EVs. Media was collected at day- 9 and kept frozen until the analysis by TEM. Day-9 blastocysts were also collected and conserved for ultrastructure analysis using TEM (See S1 Fig).

The second experiment was conceived to analyze the characteristics of the secreted EVs population and the possible correlation with embryo competence. Embryos were cultured in groups until day 7 when blastocysts were classified. From this day on, Day-7 blastocysts were individually cultured in depleted SOF media until day 9. For this, only grade I blastocysts, those with a visible blastocoel cavity, many closely aggregated cells forming a well-developed ICM and a trophoblast formed by a very well defined layer of lengthened cells were selected.

At day 9, embryos were classified as degenerated or based on developmental stage as expanded, hatching or hatched blastocysts. Culture media only from individually cultured hatched blastocysts were collected and analyzed by NTA and cytometry. The entire amount of culture media was used for the NTA determination. The culture wells that hosted the hatched Day-9 blastocysts were immediately refilled with fresh SOF media (fresh non-EVs depleted normal media) and the blastocysts were cultured individually for additional two days, until day 11 to assess their post-hatching developmental competence. No further sampling of any type was then performed on blastocysts after day 9 of culture. At day 11 and based on their growing performance upon extended culture from days 7 to 11, blastocysts were graded retrospectively as competent or non-competent at the time they reached the day 7 of development. In this way, competent blastocysts (CB) were those that reached day 11 of in vitro development with a lineal growth rate [18]; and, non-competent blastocysts (NCB) were those with arrested development from day-9 to day-11 (See S1 Fig). Finally, the characteristics (concentration and diameter) of MVs/EXs secreted by individual embryos from day 7 to Day 9 were correlated with their graded embryo competence.

### In vitro embryo production

Bovine ovaries of beef cattle were collected from Frigosur Ltda, Chillan, VIII Region–Bio-Bio located at a distance of 8 km (15 minutes trip) from our laboratory following standard procedure described by Rodríguez et al. (2008) [19]. The cumulus oocyte complexes (COCs) were *in vitro* matured (IVM) in four-well dishes (30 COCs per 500μL well) in maturation media for 20–22 h following the procedures described by Velasquez et al. (2016) [20]. The oocytes were fertilized using frozen–thawed commercial semen of a beef bull (Semex, Canada) that was previously used in other *in vitro* fertilization procedures with good blastocyst production and following the standard protocols used in our laboratory [20]. After 18 to 22 h of IVF, the presumptive zygotes were placed in 0.5 mL of TCM-Hepes + 10% of fetal bovine serum and mechanically denuded from their cumulus cells by 4 min vortexing. After that, they were washed three times before culture.

For parthenogenetically produced embryos, matured oocytes were mechanically denuded using TCM-HEPES + 0.3 mg/ml of hyaluronidase and washed three times before culture. Denuded oocytes were activated using 7% ethanol in TCM199-HEPES for 5 min, followed by a 5 h incubation in TCM199 without HEPES and supplemented with cycloheximide (10 μg/ml) and cytochalasin B (5 μg/μl) at 39°C in a 5% CO_2_ in air atmosphere.

Presumptive zygotes were *in vitro* cultured (IVC) in groups in four-well plates (30 zygotes per 500 μL well) in synthetic oviduct fluid (SOF) supplemented with 0.37 mM trisodium citrate, 2.77 mM myo-inositol, essential and non-essential amino acids (final concentration 1X), gentamycin (50 μg/ml), 3 mg/ml essentially fatty acid free BSA, 2% FBS and 10 ng/ml EGF in a balanced gas atmosphere consisting of 5% CO_2_, 5.5% O_2_ and 89.5% N_2_, 100% humidity at 39°C. Embryo selection was done at day 7 post IVF/PA considering two variables: stage of development (blastocyst and expanded blastocyst) and quality of the embryos (excellent or fair) using criteria described in IETS manual (Stringfellow and Givens, 2010). All blastocysts at day 7 were measure to compare diameter between IVF and PA-derived blastocysts.

For experiment 1, EVs depleted culture media (SOFdep) was produced by 15 h ultracentrifugation of complete media at 120,000 x g at 4°C [21]. Selected blastocysts were washed three times in SOFdep and cultured in vitro for 48 hours in groups (10 blastocyst per 500 μL well) in SOFdep. At day 9 post IVF/PA the media was collected and kept frozen at -80°C until TEM analysis. Additionally, expanded and hatched blastocyst larger than 200 μm were collected, washed and fixed in 4% paraformaldehyde and 0.25% glutaraldehyde in 0.1 M PBS at 4°C and stored until TEM analysis [22].

For experiment 2, day-7 blastocysts were removed from culture, washed three times in SOFdep and placed in a new 96 well plate (80μL of SOFdep) for individual culture for 48 hours. At day 9, the development stage of blastocysts was evaluated and culture media was collected only from hatched blastocysts. Culture media was well identified according the corresponding blastocyst and preserved individually at -80°C until NTA and cytometry analysis. Hatched day-9 blastocysts were transferred to an individual culture in 140μL of fresh SOF (conventional) for additional 48 hours following the procedures and conditions stablished in our laboratory (Velasquez et al., 2016). Embryo culture was carried out at 39°C under gas mixture (CO_2_ 5%, O_2_ 5% and N_2_ 90%).

### Isolation of extracellular vesicles from culture media

The technique of EVs isolation from embryo culture media in experiment 1, was carried out using the method described by Thery et al. (2006) [21] with modifications. The first two steps were designed to eliminate large dead cells and debris (10000 x g for 7 min) and apoptotic bodies (30000 x g for 30 min) by successive centrifugations at increasing speed. Finally, supernatant was centrifuged at 100000 x g for 3 hours to collect extracellular vesicles pellets and re-suspend them in 25 μL PBS.

In experiment 2, the individual culture media were classified according to the developmental capacity of the blastocysts at day 11 post IVF/PA: group 1 (n = 10, IVF-derived CB), group 2 (n = 10), IVF-derived NCB, group 3 (n = 10), parthenogenetic-derived CB and group 4 (n = 10), parthenogenetic-derived NCB. To individual culture media (80 μL) from each group were added 500 μL of PBS. The isolation of EVs was carried out using the protocol described by Mulvey et al. (2015) [23]. EVs were harvested from individual culture media by two-steps centrifugation: 800 x g for 10 min (elimination of cellular debris pellets) and 2000 x g for 20 min, (elimination of apoptotic body pellets). Subsequently, the recovered supernatants (500 μL) containing the vesicles (MVs/EXs) secreted by the bovine blastocyst, were analyzed by NTA. After completing this analysis, the individual culture media were grouped according to their developmental capacity: IVF-CB, IVF-NCB, PA-CB, PA-NCB. Finally, supernatants were centrifuged at 120000 x g for 90 min to concentrate EVs pellets and re-suspended in 160 μL PBS for flow cytometry analysis.

### Transmission electron microscopy analysis (experiment 1)

Transmission electron microscopy was used to identify the morphology of EVs and to identify MVB originated from intracellular compartments as precursors of exosomes. The EVs suspended pellets were deposited on formvar-carbon-coated copper grids for whole-mount preparations and subjected to TEM analysis as previously described [21,24]. Four grids for TEM analysis of isolated vesicles were prepared from each group of embryos (IVF and PA). The grids were washed and fixed in 1% glutaraldehyde in 0.1 M PBS, and then were contrasted in uranyl-oxalate solution (pH 7.0) for 5 min and methyl cellulose-UA on ice for 10 min. A total of 65 fields and images were processed using ImageJ 1.47t software.

Day-9 expanded and hatched blastocyst previously fixed were washed twice in PBS for 5 min and fixed for the second time in 4% osmium tetroxide (OsO4) and then embedded in Spurr resin. Sections of 50-to 60 nm thickness were sliced with a diamond knife and collected on 200 mesh copper grids. The grids were contrasted with 1% uranyl acetate (1 min) and lead citrate (5 min) and visualized on a Zeiss EM 900 transmission electron microscope (TEM) (ZEISS, Germany) operated at 80 to 90 kV.

### Nanoparticle tracking analysis (experiment 2)

Isolated EVs collected from blastocysts cultured individually as described (from day 7 to day 9) were recovered at day 9 and analyzed by NTA on a NanoSight NS300 (Malvern Instruments Ltd, UK), equipped with a sCMOS camera. Size distribution and concentration of nanoparticles in liquid suspension were measured using NTA. EVs were measured by assuring that there were between 20 and 100 particles per frame. Negative controls had less than 7 particles per frame. Isolated EVs were injected in continuous sample flow with a syringe pump into the sample chamber at room temperature (RT). Each sample was measured in triplicate with the same camera settings, acquisition time of 60 s and detection threshold setting of 8. The detection threshold was similar in all samples. For this analysis, a 488 nm laser beam was applied to the dilute suspension of vesicles. Data were captured and analyzed using NTA analytical software version 3.2 Dev Build 3.2.16. Graphical analysis shows particle size distribution of the nanoparticles in the groups and concentration was reported as particles per milliliter. Multivariate cluster analysis was performed to identify similar patterns of EVs populations secreted by CB versus NCB.

### Analysis of exosome markers

For this analysis, culture media collected from individual embryos in Experiment 2 was first individually analyzed by NTA and then recovered and pooled for flow cytometry. At least 3 pools of 3 embryos form each experimental group were considered (IVF-CB, IVF-NCB, PA- CB, PA-NCB). Due to their small size, EVs cannot be used directly in flow cytometry characterization, instead they were bound to latex beads as described earlier [21]. Briefly, EVs (35 μL or 4 x 10^8^ particles/ml) were incubated with 4 μm aldehyde/sulfate latex beads (0.125 μl or 1.25 x 10^5^ particles/ml) (Life Technologies, Santiago, Chile) in a 100 μl final volume of PBS and incubated overnight at 4°C. Next, 22 μL of 1 M glycine/PBS (100 mM final concentration) was added and mixed gently (to block the unbound sites of the latex beads) and kept for 45min at RT. MVs/beads complex were washed twice with 1 mL of PBS/0.5% bovine serum albumin by centrifugation at 1500 x g for 3 min at RT. The MVs/beads complexes were incubated with primary FITC-conjugated anti-human antibodies against CD63 (Abcam, catalog no. 18235, clone MEM-259) or CD9 (Abcam, catalog no. 34162, clone MM2/57) for 1 hour at RT. A negative control antibody reaction was performed using latex beads alone incubated with anti-CD63 or anti-CD9 for 1 hour at RT. Exosomes from a commercial human embryonic kidney cell line (HEK293; Life Technologies, Grand Island, NY, USA) were used as positive controls. The labelled MVs/beads complex were pelleted and washed twice as above with 1mL of PBS/0.5% bovine serum albumin, and finally 100 μL pellets were resuspended in 400 μL of focusing fluid and subjected to flow cytometry using Attune^™^ NxT Flow Cytometer (Life Technologies, Inc, USA).

### Statistical analysis

In the first experiment, for comparing the rate of embryonic development at day 7 and at day 9, two proportions statistic test was used. Blastocyst diameter on day 7 and day 9 was compared using t-student statistic test for independent samples.

In the second experiment, we used NTA and FACS analysis to identify and characterize the CB considering the characteristics of the vesicles secreted in the media culture from day 7 to 9. Descriptive statistics were computed for each group. The differences between the 4 groups (IVF-CB, IVF-NCB, PA-CB and PA-NCB) were assessed by a one-way analysis of variance (ANOVA) with Tukey's studentized range (HSD) test or t-tests (LSD) for multiple comparisons between means. Significance was set at p<0.05. Data of particle concentration by size from NTA analysis was evaluated using principal components analysis (PCA) and cluster analysis (CA) procedure through PROC PRINCOMP and PROC CLUSTER (Average Linkage Cluster Analysis) to differentiate grouping of data with similar characteristics. Statistical analysis was performed with the program the SAS system version eight for windows (SAS Institute Inc., Cary, NC, USA, 1999).

## Results

### Exp. 1. Identification of EVs and MVB

In this experiment, embryos were cultured in groups, excellent blastocysts were selected at day 7 and grouped in tens in depleted media until day 9 of development. The rates of blastocysts at day 7 and of expanded blastocysts at day 9 are shown in Table 1 and S2 Fig. No statistical differences in the blastocyst rate, embryo size (diameter) and expanding rate at day 7 were observed between the two groups (PA and IVF). Nevertheless, at day 9 of culture, expanded blastocyst derived from IVF had a significantly higher average diameter than those derived from PA (Table 1).

**Table 1:** Comparison between IVF and PA blastocyst at days 7 and 9 of development.

Group	COCs n	Blastocyst (Day 7) n (%)	Cultured in exosome-depleted media Blastocyst (Day 7) to Expanded blastocyst (Day 9)			
			n	diameter, μm (x±sd)	n (%)	diameter, μm (x±sd)
**PA**	215	77 (35.8%)	63	183.3±43.2	33 (52.4%)	274.4±51.8^a^
**IVF**	216	63 (29.2%)	60	197.2±38.8	33 (55.0%)	338.3±95.3^b^
**P value**		0.15		0.17	0.85	0.02

Values in the same column carrying different superscripts are considered statistically significant at p < 0.05. COCs: cumulus oocyte complexes.

**Fig 1:**
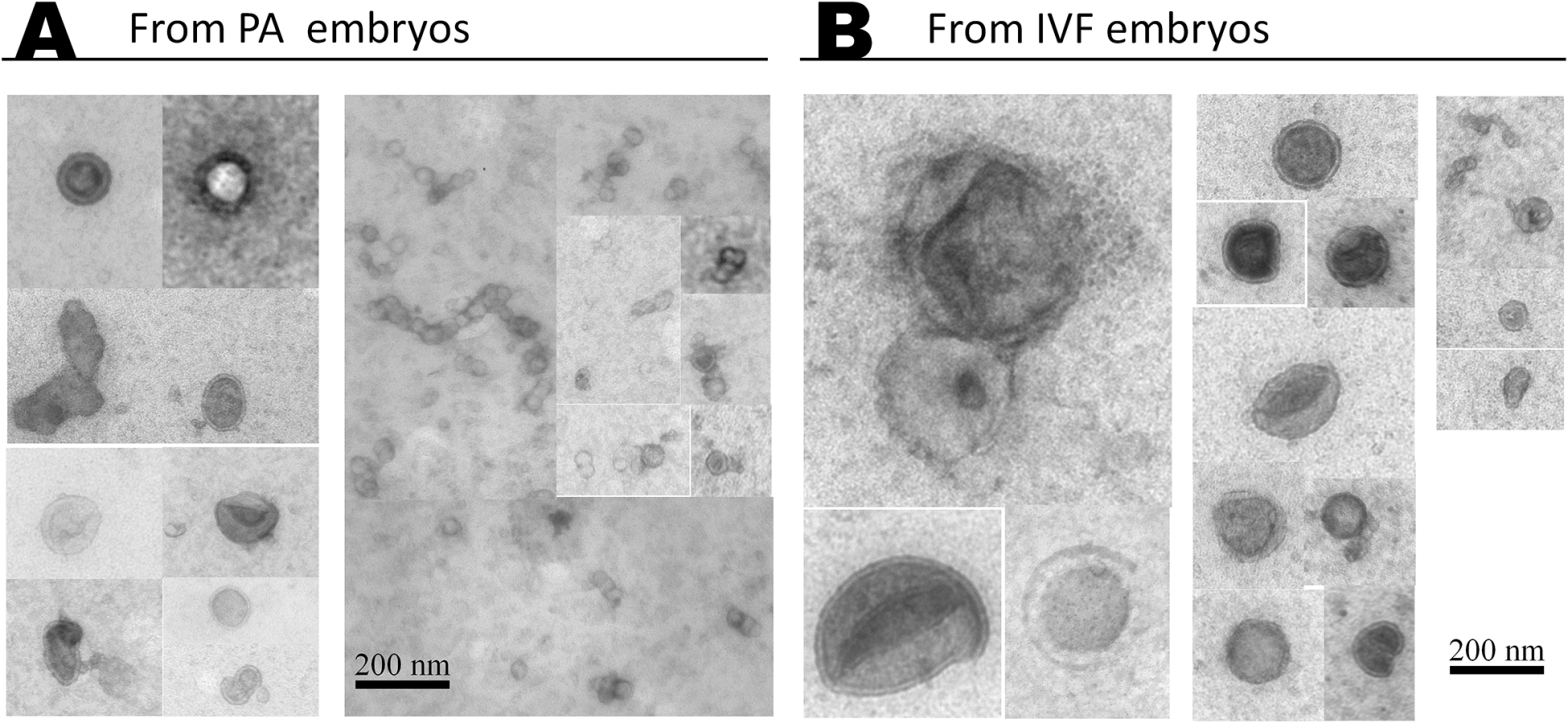
Morphology of EVs secreted by bovine blastocysts. Representative pictures from transmission electron micrographs showing EVs secreted to culture media by IVF (B) and partenogenetic (A) derived blastocysts from day 7 to 9 of in vitro development.

TEM analysis of the EVs secreted by blastocysts during culture from day 7 to day 9 showed the presence of at least two populations of EVs: MVs and EXs with a characteristic dense appearance and rounded shape enclosed by lipid bi-layer and with a diameter ranging from 30 to 385 nm (Fig 1A and 1B).

**Fig 2:**
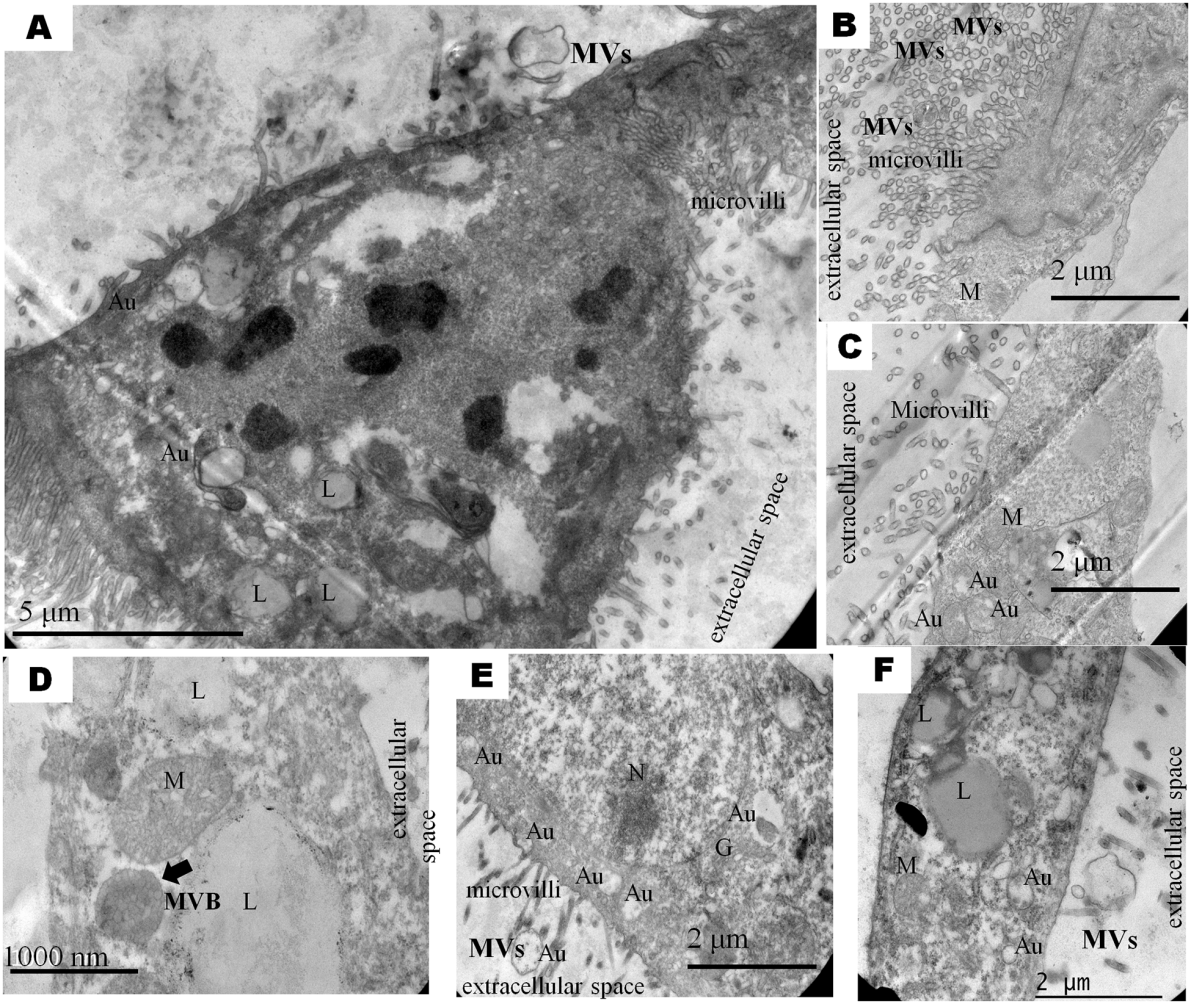
Representative microphotograph showing ultrastructure of bovine IVF and PA derived blastocysts. Nucleus (N; in E), mitochondria (M; in C, D, F), lipids (L; in A, D, F), autophagosome (Au; in A, C, E, F), multivesicular body MVB (arrow in D) and microvilli (in A, B, C, D, E) shown superficially of the cell.

TEM allowed for clear localization of different organelles, such as mitochondria, Golgi apparatus, MVB, autophagosome and lysosomes in blastomeres (Fig 2). A marked and conspicuous presence of large lipid droplets in both PA and IVF derived blastocysts was observed. Also, there were numerous microvilli projected into the space between the blastomeres, but they appeared to be shorter and less developed in IVF embryos than in PA embryos.

### Exp. 2. Characteristics of the EVs population secreted CB and NCB

After identifying EVs secreted by pre-implantation embryos, in a second and independent experiment we studied the possible impact of developmental competence of blastocysts on the concentration of EVs secreted from day 7 to 9 of individual in vitro culture. In this experiment, embryos were cultured until day 7 when blastocyst were selected and placed individually in a new dish with depleted SOF media. Blastocysts were culture for two days (until day 9). At day 9, culture media was collected to study MVs, blastocysts diameter was measured and blastocyst were kept in individual culture in fresh normal SOF media for two additional days (until day 11).

**Fig 3:**
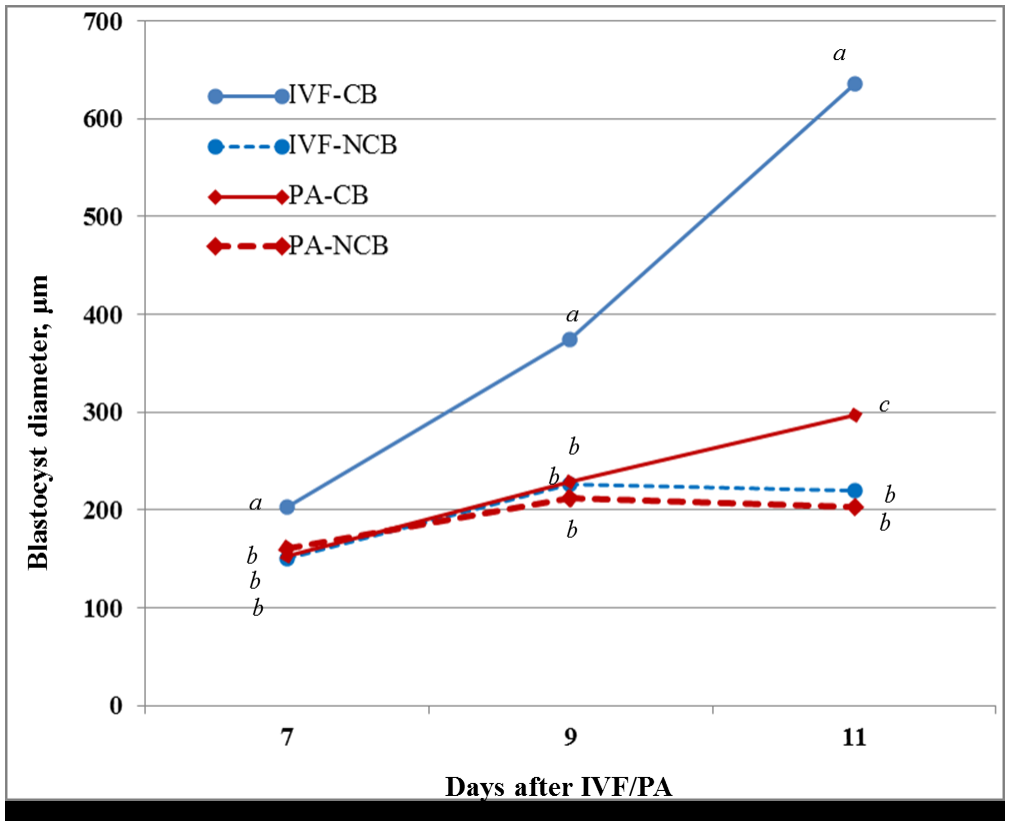
Growth rate of blastocysts cultured in vitro according to their competence from day 7 to 11. Values at the same day of embryo development carrying different superscripts are considered statistically significant at p < 0.05. CB: Competent blastocysts; NCB: non-competent blastocysts.

Growth rate of the blastocysts derived from IVF and PA that were classified as CB and NCB from day 7 to 11 is shown in Fig 3 and S1 Table. CB derived from IVF had the highest diameter (663.1 μm) at day 11 of in vitro development (p< 0.05), compared to CB derived from PA of the same age (297.8 μm). NCB showed similar growth rate and size in spite of their origin (IVF or PA). Although, blastocysts derived from parthenogenic activation at day 7 and 9 did not show significant differences in their diameter between competent and non-competent (see Fig 3 and S1 Table).

### Characterization of EVs populations

**Fig 4:**
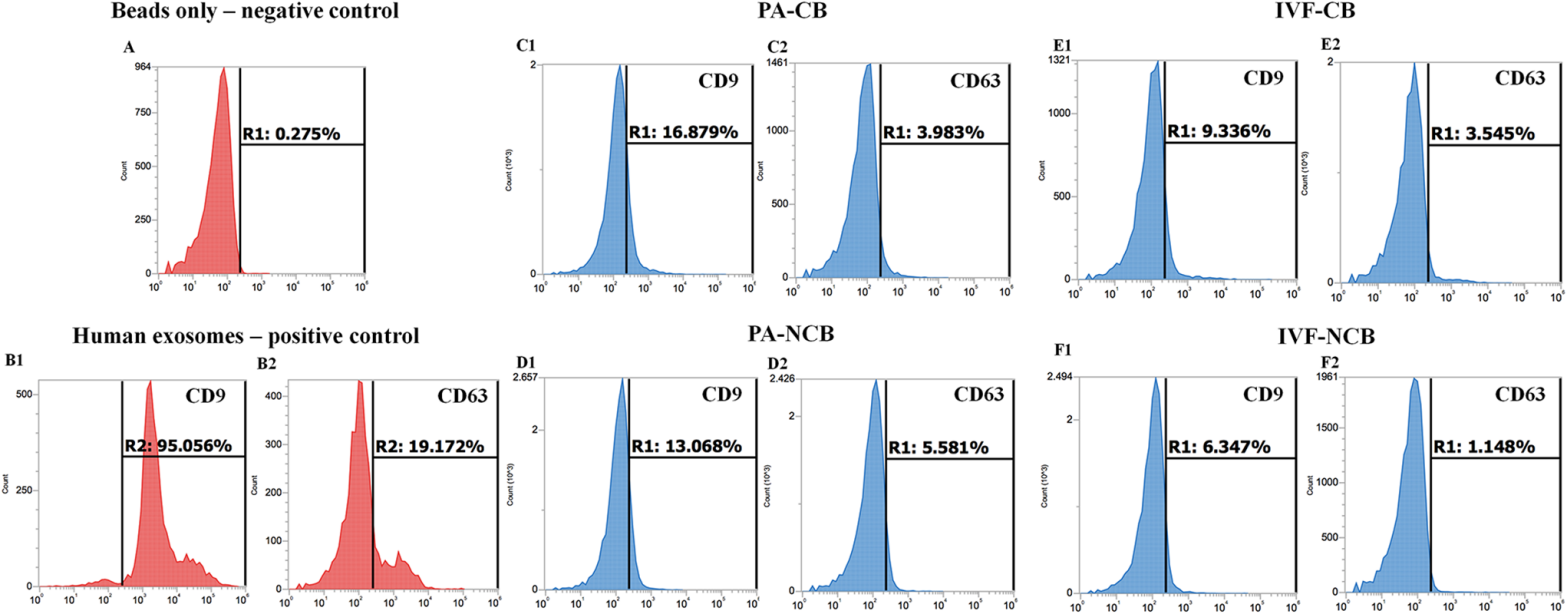
Flow cytometry analysis of EXs markers (CD9 and CD63). Negative control-beads alone (A); positive control–EXs isolated from human embryonic kidney (HEK293) (B); EXs in competent (C and E) and non-competent (D and F) blastocysts.

Flow cytometry determined the presence of specific surface markers for EVs. Due to the small size of EVs, they were conjugated with latex beads of 4 μm and surface markers were assessed using fluorescent antibodies against the tetraspanins CD9 and CD63. Both, CD63 and CD9 proteins were identified in EVs secreted from IVF and PA blastocysts (Fig 4), though CD63 was barely detected. The positive population to CD9 was higher compared to CD63 in both IVF and PA blastocysts. Also, the positive control showed a higher population of EXs to CD9 marker (97.7% CD63 vs 13.2% CD9). The negative control showed less than 0.3% positivity.

**Fig 5:**
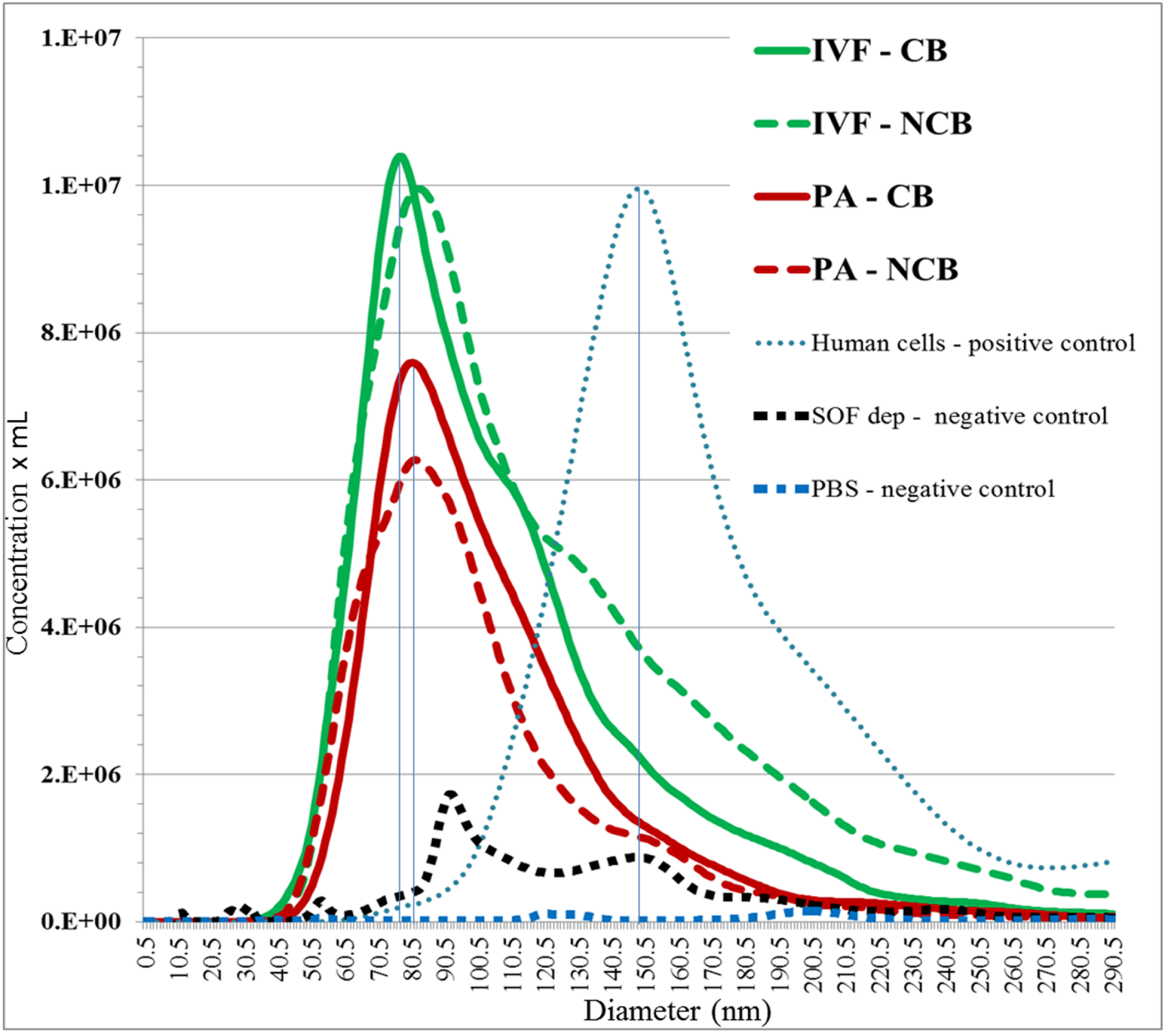
Tracking of nanoparticles to determine size distribution of EVs populations from IVF and PA blastocysts, according to their competence. The horizontal bars indicate the exosome size range. The EVs depleted SOF media and PBS used to resuspend isolated EVs, were included as negative controls. As a positive control, collected culture media from human cells was used.

NTA analysis was performed in culture media from individual blastocysts from day 7 to 9 of in vitro development. The distribution of size of EVs ranged from approximately 17 to 300 nm in diameter (Fig 5). EVs from IVF and PA blastocysts exhibited similar statistical size descriptors (media and mode), only concentration was different (Table 2).

**Table 2:** Statistical descriptors for EVs populations secreted by blastocysts according to their competence until day 11.

Group	n	EVs Size		Concentration (x10^8^ particle/mL) (x ± sd)
		Media, μm (x ± sd)	Mode, μm (x ± sd)	
**IVF-CB**	10	107.3 ± 12.9	75.1 ± 7.3	6.7 ±3.1 ^ab^
**IVF-NCB**	10	122.0 ± 28.7	78.9 ± 13.5	8.5 ± 3.7 ^a^
**PA-CB**	10	107.0 ± 13.3	83.4 ± 10.6	4.7 ± 1.3 ^b^
**PA-NCB**	10	104.5 ± 18.4	81.3 ± 16.2	3.9 ± 1.2 ^b^

Values in the same column carrying different superscripts are considered statistically significant at p < 0.05. CB: competent blastocysts; NCB: non-competent blastocysts. Culture media from 10 individual embryos was analyzed in each group.

**Fig 6:**
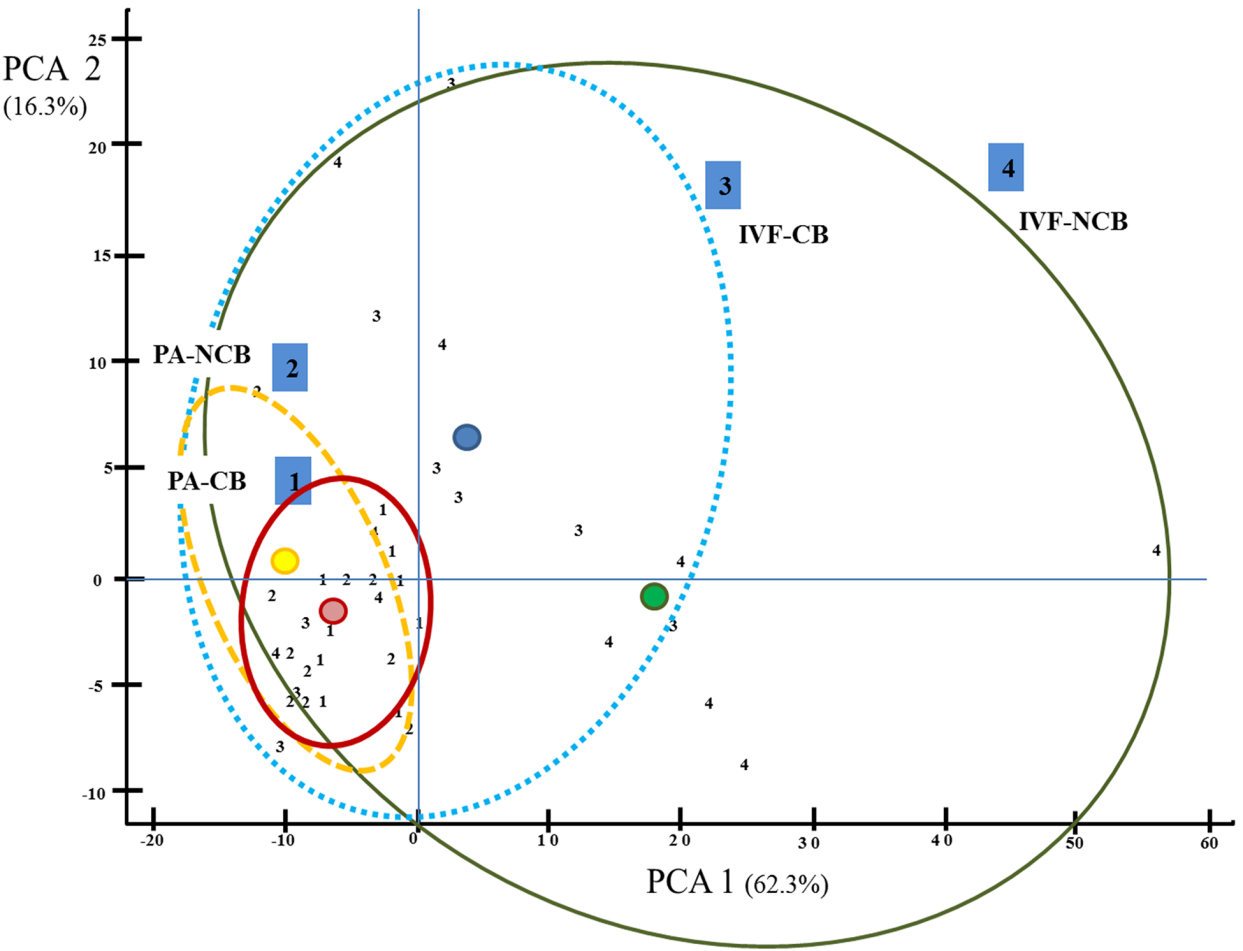
Hierarchical Cluster Analysis (CA) of the first two principal component axes (Prin1 and Prin2) for EVs concentration according to vesicle size showing clusters based on blastocysts competence. Each number represents the concentration of EVs from individual embryo from each experimental group: Number 1: Represents individual embryos from PA-CB; Number 2: Individual embryos from PA-NCB; Number 3: Individual embryos from IVF-CB; Number 4: Individual embryos from IVF-NCB.

The population of EVs secreted by blastocysts was analyzed using multivariate cluster analysis to identify the major trends of groups between CB and NCB with different origin (IVF and PA). The first two principal components (prin 1 and prin 2) account for over 78.6% of the total variance in either dataset (Fig 6). Each cluster is including the concentration of EVs from individual embryos from the same experimental group based on embryo origin and competence (1: PA-CB; 2: PA-NCB; 3: IVF-CB; 4: IVF-NCB). The size of the cluster is related with the dispersion of the data within the group. Table 2 and Fig 6 show that the population of EVs from IVF/PA blastocysts is different in concentration. The population of EVs from PA embryos is more homogeneous compared to IVF-derived embryos. However, the analysis of EVs population secreted by IVF embryos suggests a higher variability in embryos classified as non-competent (IVF-NCB).

## Discussion

To the best of our knowledge, this is the first study showing secretion of EVs by bovine in vitro cultured embryos. In this research, it was possible to isolate and morphologically characterize embryo-secreted EVs. Using TEM, we found two types of EVs: MVs and EXs enclosed by lipid bi-layer membranes ranging from 30 to 385 nm of diameter. In agreement with our results, Saadedin et al. (2014) [13] observed, in culture media of day-7 parthenogenic porcine embryo, EVs with a size range of 30 to 120 nm. This difference in size could be due to the embryos being of different species, and also to the zona pellucida present in day-7 blastocysts, which might potentially regulate the size of the particles released to the extracellular environment. Most of the day-7 to day-9 blastocysts here, were released from the zona pellucida which could explain the higher range of EVs size observed. Although, we did not see differences in morphology of EVs derived from PA and IVF blastocysts.

Additionally, we identified EVs and MVB structures that indicate that the trophoectoderm cells secrete EVs. Fader et al. (2008) [17] affirmed that MVB reoriented from the autophagic pathway release EXs. MVB are well characterized as endosomal precursors of the lysosomal degradation pathway and can also fuse with the plasma membrane to release EXs [25]. Therefore, the identification of MVB and autophagosomes in the ultrastructure embryonic cells might indicate that autophagic machinery and EXs secretion are present in bovine embryos.

Autophagy is the main physiological pathway for the degradation of intracellular macromolecules, it plays a specific function in the turnover of organelles, proteins and RNA [26,27] and have essential roles in early embryonic development [28]. Furthermore, autophagy induction contributes to increase cell survival, presumably through the selective removal of late endosomes by the autophagy machinery [15,16,17]. So, this suggests that embryonic cells in apoptosis or programmed cell death may influence the amount of released EXs. This is coincident with our results from NTA analysis indicating that the concentration of EVs is higher in NCB derived from IVF.

Besides morphological characterization, it is strongly desirable to identify specific surface markers as confirmative criterion of EVs. Flow cytometry is a robust tool for reliable detection of cell surface proteins, and has many advantages over other immunological techniques, such as Western blotting and immunohistochemistry [29].

Although, the ISEV suggests authors to include transmembrane proteins (Tetraspanins CD9, CD63, CD81) and cytosolic proteins with membrane-binding capacity (TSG101, annexins = ANXA, Rabs = RAB) in their publications [8]; none of these proteins are exclusive to EXs neither are specific markers to distinguish EVs subtypes. However, due to the abundance of these proteins in extracellular vesicles, they are used as exosomal markers [30]. Our flow cytometry analysis for CD9 and CD63 showed the presence of both proteins in EVs secreted from day 7 to 9 of the in vitro culture of IVF and PA derived blastocysts as well as in positive controls. However, only a small population of EVs-beads complex was positive to any of the markers in experimental groups. This can be explained by non-abundant linkage of EVs to beads. Furthermore, a higher proportion of EVs-beads complex in the positive control marked stained for CD9 (97.7%) compared to CD63 (13.3%), this pattern have been also observed in the analysis of the EVs secreted by bovine blastocysts in our experiments, as well as by other cell types (data not shown).

Data from NTA showed that the profile (concentration and diameter) of secreted particles by embryos derived from IVF is different (not statistically) from the population of vesicles secreted by PA embryos. Ferreira et al. (2013) [14] indicated that average EVs size secreted by human embryos could be associated with decreasing embryo quality and proposed that 202nm is predictive of good embryos, while 218nm is characteristic of average embryos, 222nm of poor and 227 nm of slow development). Saadedin et al. (2014) [13] found an association between the vesicle size and the state of embryonic development (EVs <40nm in the two-cell stage until porcine blastocyst formation and <120 nm from the blastocyst stage expansion until hatching).

Based on their results, Saadedin et al. (2014) [13] suggested that size distribution of EVs secreted by pre-implantation embryos could be influenced by embryo stage while Ferreira et al. (2013) [14] proposed a relation with embryo quality. In this work, we did not find statistical differences between EVs populations from IVF and PA derived embryos. When multivariate cluster analysis was used to identify the major trends among groups of competent or non-competent blastocysts, coming from different origins (IVF and PA: Fig 6), we found that the distribution of EVs populations had a higher variability in non-competent embryos; this might be indicative that the secretion of EVs is influence by embryo origin and competence.

The findings presented here about the secretion of EVs by bovine embryos could be indicative of the cell status and could be useful for selecting competent blastocyst. However, advanced studies on EVs content is needed for a better understanding of their role and a possible use as a non-invasive method for embryo assessment and selection. Furthermore, the ultimate criterion for assessing EVs as predictive tool of embryo quality and developmental capability is development to term and calving. Nevertheless the work presented here is the first approach toward that goal. Further experimental confirmation is still needed as to compare with embryos produced from other biotechnologies such as nucleus transfer, or ICSI.

## Conclusions

We demonstrated that bovine blastocysts secrete MVs/EXs to the culture media and that the concentration of EVs population secreted to the culture media from day 7 to day 9, vary depending on embryo competence and origin.

## Supporting information

S1 Fig Experimental design. For both experiments, embryos derived from in vitro fertilization (IVF) or parthenogenetic activation (PA) were cultured in group until day 7; excellent blastocysts were selected and cultured in depleted media in group of 10 blastocysts (Experiment 1) or individually (Experiment 2) up to day 9. At day 9, embryo culture media was collected. In Experiment 1, collected media from each group of 10 blastocysts was used for TEM analysis as well as good quality Day-9 blastocysts. In experiment 2, culture media from hatched blastocysts was collected individually and analyzed by NTA and cytometry). Blastocysts were put back for individual culture in SOF media (fresh normal media) until day 11 in culture to assess developmental competence.
(TIF)

S2 Fig (A) Parthenogenic- and (B) IVF-derived blastocysts (Day, 9) after culturing in SOFdep for 48h. (C) Spherical shape of the embryo between day 7 and 11 and after day 11 post-fertilization in vitro.
(TIF)

S1 Table Characteristics of blastocysts cultured in vitro according to their competence at day 11. (DOCX)
