# Supplementary figures and images for: Identification and characteristics of extracellular vesicles from bovine blastocysts produced in vitro
Source: PLoS One. 2017 May 25;12(5):e0178306. doi: 10.1371/journal.pone.0178306 (PMC5444795; doi:10.1371/journal.pone.0178306)

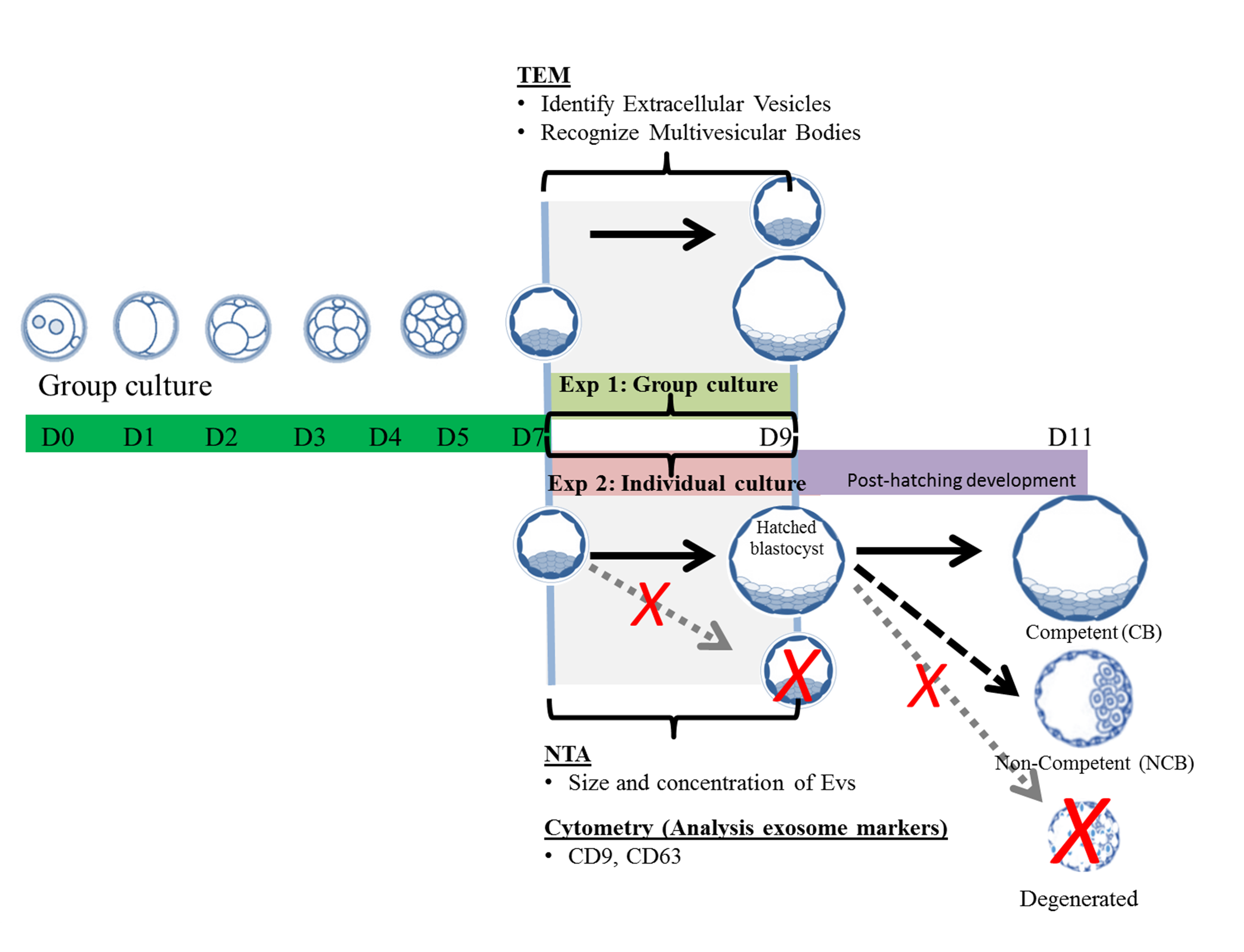

Supplement: S1 Fig — For both experiments, embryos derived from in vitro fertilization (IVF) or parthenogenetic activation (PA) were cultured in group until day 7; excellent blastocysts were selected and cultured in depleted media in group of 10 blastocysts (Experiment 1) or individually (Experiment 2) up to day 9. At day 9, embryo culture media was collected. In Experiment 1, collected media from each group of 10 blastocysts was used for TEM analysis as well as good quality Day-9 blastocysts. In experiment 2, culture media from hatched blastocysts was collected individually and analyzed by NTA and cytometry). Blastocysts were put back for individual culture in SOF media (fresh normal media) until day 11 in culture to assess developmental competence. (TIF) [file pone.0178306.s001.tif]

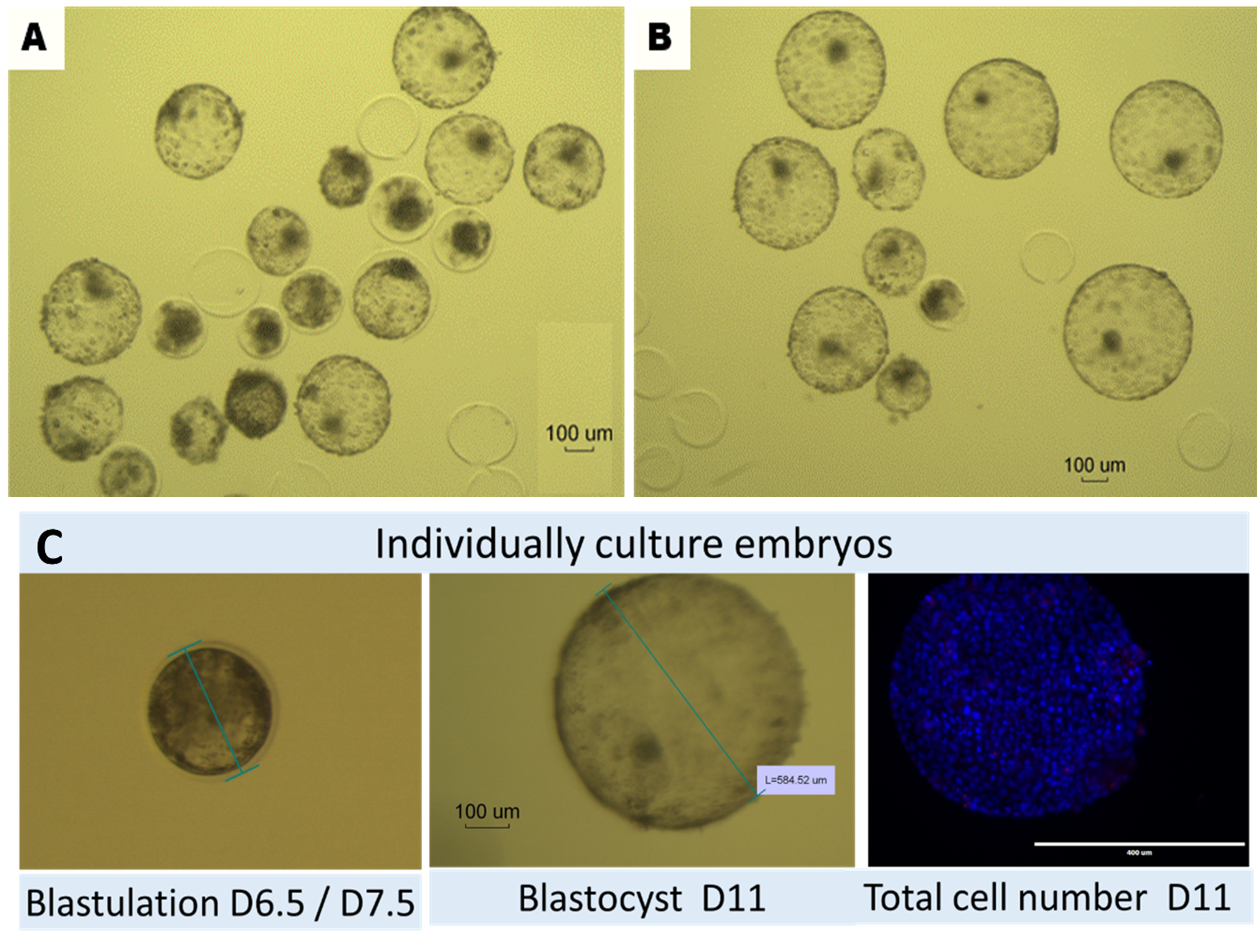

Supplement: S2 Fig — (A) Parthenogenic- and (B) IVF-derived blastocysts (Day, 9) after culturing in SOFdep for 48h. (C) Spherical shape of the embryo between day 7 and 11 and after day 11 post-fertilization in vitro. (TIF) [file pone.0178306.s002.tif]
