# Supplementary material for: Identification and characteristics of extracellular vesicles from bovine blastocysts produced in vitro
Source: PLoS One. 2017 May 25;12(5):e0178306. doi: 10.1371/journal.pone.0178306 (PMC5444795; doi:10.1371/journal.pone.0178306)
Supplement: S1 Table — (DOCX) [file pone.0178306.s003.docx]

**S1 Table.** Characteristics of blastocysts cultured in vitro according to their competence at day 11.

| \| **Group** \| **n** \| **Day-7 (µm)** \| **Day-9 (µm)** \| **Day-11 (µm)** \| \| --- \| --- \| --- \| --- \| --- \| \| **IVF-CB** \| 10 \| 203.4 ± 19.1 ^a^ \| 374.1 ± 74.1 ^a^ \| 636.0 ± 134.0 ^a^ \| \| **IVF-NCB** \| 10 \| 150.6 ± 19.0 ^b^ \| 226.9 ± 28.4 ^b^ \| 219.8 ± 42.4 ^b^ \| \| **PA-CB** \| 10 \| 153.9 ± 22.0 ^b^ \| 228.7 ± 33.4 ^b^ \| 297.8 ± 71.7 ^c^ \| \| **PA-NCB** \| 10 \| 160.9 ± 21.2 ^b^ \| 213.3 ± 31.6 ^b^ \| 203.2 ± 16.3 ^b^ \| |
| --- | --- | --- | --- | --- | --- | --- | --- | --- | --- | --- | --- | --- | --- | --- | --- | --- | --- | --- | --- | --- | --- | --- | --- | --- | --- |

Values in the same column carrying different superscripts are considered statistically significant at p < 0.05.
